# Histological fidelity and microenvironmental kinome signatures of metastatic patient-derived organoids

**DOI:** 10.3389/fbioe.2026.1735980

**Published:** 2026-04-02

**Authors:** Joana Leitão-Castro, Alexia Melanie Lopresti, Kamilla Westarp Zornhagen, Reidar Albrechtsen, Juan Camacho-Roda, Mille Bylov Ravn, Alejandro Gutierrez Martinez, Luis Arnes Perez, Eric Santoni-Rugiu, Martin Højgaard, Kristoffer Staal Rohrberg, Ulrik Lassen, Janine Terra Erler

**Affiliations:** 1 Biotech Research and Innovation Centre (BRIC), University of Copenhagen (UCPH), Copenhagen, Denmark; 2 Department of Oncology, Rigshospitalet, Copenhagen University Hospital, Copenhagen, Denmark; 3 Department of Clinical Medicine, University of Copenhagen, Copenhagen, Denmark; 4 Department of Pathology, Rigshospitalet, Copenhagen University Hospital, Copenhagen, Denmark

**Keywords:** kinome, metastasis, organoids, precision medicine, tumour microenvironment

## Abstract

Metastatic cancer remains the greatest clinical challenge due to ineffective treatments and limited reliable models for drug testing. Patient-derived organoids (PDOs) and their subsequent culture as patient-derived-organoid xenograft (PDOX) tumours have transformed cancer research by replicating the genetic and histological characteristics of primary tumours. However, less focus has been given to metastatic tumours. Here, we investigated how well kinase signalling was conserved in PDOs grown from core-needle biopsies isolated from metastatic tumour lesions of five cancer patients. We further compared changes in kinase signalling when these PDOs were grown as metastatic PDOX tumours in mice. Strikingly, PDOs retained much kinase signalling observed in the original tumour. Even more remarkable was the ability of the metastatic PDOX tumours to match both the signalling and morphological features of the original biopsy. Cross-sample analysis revealed lost Src Family Kinase signalling in PDO cultures, highlighting the important influence of the tumour microenvironment on signalling and demonstrating this can be partially restored in the *in vivo* setting. Together, these studies support the use of PDOs and derived PDOX in mimicking and modelling human metastatic tumour biology.

## 1 Introduction

One of the greatest challenges of translational medical research and personlised medicine approaches is the lack of reliable models that mimic and recapitulate the complexity of the patient’s tumours. The current state of precision oncology heavily relies on preclinical models that accurately recapitulate patient-specific tumour biology (Bose et al., 2021; Wang et al., 2022; Tong et al., 2024).

Traditional two-dimensional approaches, often fail to retain the complexity of these systems, lacking cellular and structural heterogeneity of tumours. The development of more complex *in vitro* approaches, such as patient-derived organoids (PDOs), has presented a major leap in oncological research (Bose et al., 2021; Wang et al., 2022; Tong et al., 2024). Most research has focused on PDOs isolated from the primary tumour and has demonstrated that PDOs retain some of the features and heterogeneity of the patient tumours, and can therefore assist in selecting the most appropriate therapy for patients (Wang et al., 2022; Tong et al., 2024; Ogawa et al., 2025). However, little is known about how well PDOs from metastatic tumours recapitulate the biology of their original tumours both *in vitro* and *in vivo* (Vlachogiannis et al., 2018).

Protein kinases form a densely interconnected signalling network, the kinome, that integrates growth factor, adhesion, stress, and immune cues to control proliferation, survival, migration, and differentiation in tumour cells (Creeden et al., 2020; Katoh, 2024a). Dysregulation of this network is a hallmark of cancer and underpins both oncogenic signalling and adaptive resistance to therapy, as illustrated by the clinical success of multiple kinase inhibitors across solid and haematological malignancies (Al-Ejeh et al., 2014; de Bous et al., 2020; Fabro et al., 2022). Systematic kinome profiling directly captures the functional activity state of these pathways, enabling identification of actionable signalling vulnerabilities, prediction of drug sensitivity, and discovery of resistance mechanisms that are not apparent from genomic or transcriptomic data alone. Accordingly, kinome-based signatures and indices have shown prognostic value and can refine risk stratification and guide selection of targeted therapies in several cancer types (Al-Ejeh et al., 2014; Duong-Ly and Peterson, 2013; Radu and Chernoff, 2017; Yao et al., 2024).

To address this, and to further understand the reliability of these models to further study cancer progression and therapeutical options, we established PDO lines from five different patients’ metastatic tumour biopsies and engrafted them into NSG mice. Metastatic tumours were harvested from mice and compared histologically to the original patient metastatic tumours through blinded pathological analysis. We further performed kinase profiling across the three sample types: the original patient metastatic tumour biopsy (KP1), the matched PDO (KP2), and the grafted metastatic tumour (KP3), to investigate potential alterations induced by the *in vitro* context, and assess signalling dynamics in these different contexts.

Strikingly, we found KP3 tumours retained the histopathological features of their original metastatic tumour biopsies (KP1), underscoring their potential as accurate *in vivo* models. Interestingly, the observed kinase alterations–invisible in the KP2 cultures–were primarily restricted to signatures associated with the tumour microenvironment, suggesting that while core tumour features are preserved across the samples, tumour microenvironment remains a key feature that should not be neglected in these models and potential therapeutic decisions. Nonetheless, these findings highlight the power of PDOs in accurately recapitulating human patient disease *in vitro* and as PDOXs *in vivo*, across different cancers, in particular the value of engrafted PDO models, not only for personalised medicine approaches and translational research, but also for undercovering clinically relevant, context-dependent signalling networks, that should be taken into account to guide therapeutic development.

## 2 Materials and methods

### 2.1 Patient-derived organoid generation

Patient-derived organoid (PDO) lines were established from 18G core-needle biopsies isolated from metastatic tumours of cancer patients, who provided informed consent approved by the Ethics Committee of the Capital Region of Denmark (approval no. H-16046103, Data Registry ref: 2012-58-004). Briefly, biopsy tissue fragments were finely minced and processed for organoid culture as previously described (Kjølle et al., 2026; Rafaeva et al., 2022). Cultures were maintained for up to 6 months, with successful patient-derived organoids (PDOs) defined by organoid-like morphology, robust passaging capacity. For passaging, BME domes were resuspended in ice-cold PBS (Sigma-Aldrich), pelleted (1,400 rpm, 5 min at 4 °C), and mechanically disrupted by pipetting prior to resuspension in fresh BME for replating. All assays used low-passage organoids (≤ passage 10), which were routinely confirmed Mycoplasma-free.

### 2.2 Intrasplenic injections

8-week-old female NSG (NOD.Cg-Prkdcscid Il2rgtm1Wjl/SzJ) mice (Taconic Biosciences, NY, USA) were maintained under aseptic conditions, in an appropriate pathogen-free facility. Intrasplenic injections were performed as previously described (Nielsen et al., 2021). Briefly, mice were placed under anaesthesia with isoflurane (2%–3% in oxygen) and analgesics administered. A small incision was made on the left flank to exteriorise the spleen.

For injection, PDOs were dissociated into single cells and resuspended in PBS. A suspension of 5x10^5^ cells in 10 µL of PBS was injected into the splenic parenchyma using a 30G Hamilton Gastight 705 RN Syringe. Upon injection, gentle pressure was applied to prevent reflux of the cells, and the spleen was returned to the abdominal cavity. The peritoneum and skin were closed with absorbable sutures. Mice received postoperative analgesia and were monitored regularly. All procedures were conducted under sterile conditions, following protocols approved by the Danish Animal Experiments Inspectorate (permission number #2020-15-0201–00596).

Livers were surgically resected upon reaching the humane endpoint. Pieces of the tumour were snap frozen for further protein collection and the remaining liver fixed in formalin for further histological sectioning.

### 2.3 Histological analysis

Formalin-fixed liver samples were embedded in paraffin and sectioned for routine H&E staining, as previously described (Nielsen et al., 2021). Single sections were analysed by a blinded Board-Certified pathologist.

### 2.4 Pamgene kinase profiling

Kinase profiling of tissue samples was performed as previously described (Rafaeva et al., 2022). Briefly, snap frozen mouse tumour samples were homogenised in M-PER™ (Mammalian Protein Extraction reagent) supplemented with 1% Halt™ phosphatase inhibitor cocktail and 1% Halt™ protease inhibitor cocktail (ThermoFisher, Massachusetts, USA), using a tissue homogeniser, followed by 1 hour incubation at 4 °C at 500rpm. Samples were centrifuged at x16000 g for 10 min, and supernatant was collected. For core-needle biopsies from metastatic tumours, protein was extracted by sterile cutting the biopsy into small pieces, placing these into M-PER™ supplemented with 1% Halt™ phosphatase inhibitor cocktail and 1% Halt™ protease inhibitor cocktail (ThermoFisher), flicking bottom of tube to start lysis and incubating for 30 min at 4 °C, followed by centrifugation and supernatant collection. PDO samples were harvested using ice cold PBS and washed several times with ice cold PBS to remove BME before resuspending the pellet in M-PER™ supplemented with 1% Halt™ phosphatase inhibitor cocktail and 1% Halt™ protease inhibitor cocktail (ThermoFisher) for lysis in the same way as described for core-needle biopsies. Protein concentration in the samples was quantified by Bradford analysis.

Pamgene assays and analysis were performed following manufacturer’s instructions (Pamgene-Hertogenbosch, Netherlands). 5µg of protein were used for the PTK assay. Data analysis was performed using the Tercen BioNavigator Platform. Raw data was processed and plotted in GraphPad Prism 10.5.0.

### 2.5 ELISA

Phospho-Src (Tyr416) levels were quantified using the PathScan® Phospho-Src (Tyr416) Sandwich ELISA Kit (Cell Signaling Technology #7953) following the manufacturer’s protocol. Cell lysates were added to antibody-coated microplates and incubated with total Src detection antibody followed by HRP-conjugated secondary antibody. After TMB substrate development and addition of STOP solution, absorbance was measured at 450 nm on a microplate reader. Optical density (OD) values from the measured absorbance were normalised to KP3 samples (set to 1) within each matched patient set, with KP2 values expressed as fold-change relative to KP3.

### 2.6 Data availability statement

All data generated or analysed during this study are included in this published article and its Supplementary Material files.

## 3 Results

### 3.1 PDO-derived tumours retain patient-specific tumour histology

**FIGURE 1:**
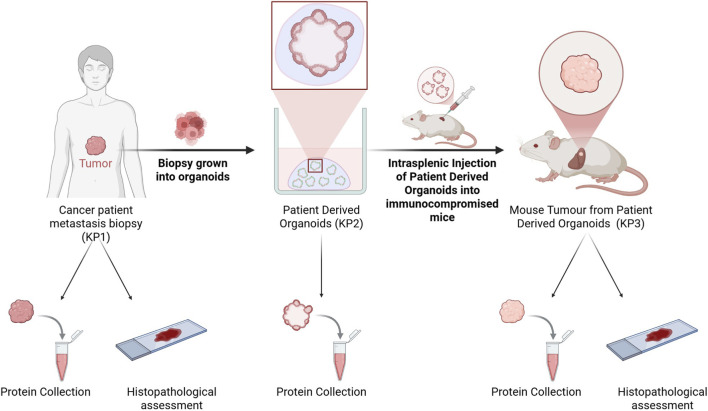
Experimental workflow for generation and collection of samples. Patient metastatic tumour samples (KP1) were obtained from metastatic tumour biopsies of cancer patients, and further processed for both proteomic and histopathological analysis. A portion of the biopsy tissue was dissociated and cultured to establish patient derived organoid (PDO) cultures (KP2), which were used as *in vitro* models of the original tumour metastasis, and used to further collect protein. These PDOs were then injected as a single cell suspension into the spleen of immunocompromised NSG (NOD.Cg-Prkdcscid Il2rgtm1Wjl/SzJ) mice. Once mice reached a humane endpoint, they were euthanised and their livers collected. A portion of the tumour was used to extract protein, and the remaining liver was sectioned and used for histopathological assessment (KP3).

As a way to model patient tumours in both *in vitro* and *in vivo* settings, and assess the robustness of PDOs and PDO-derived tumours as pre-clinical models, we established a workflow that enabled integrated histological and proteomic analysis across multiple biological contexts (Figure 1). Tumour biopsies from metastatic lesions were isolated from five cancer patients and processed in parallel for proteomic and histopathological assessment (KP1). Histological assessment classification can be found on Table 1. A portion of each biopsy was dissociated and cultured to generate patient-derived organoids (PDOs), which served as *in vitro* tumour models (KP2). PDO cultures were expanded for 1–4 months and harvested for kinase profiling, and in parallel grafted into the spleens of NSG (NOD.Cg-Prkdcscid Il2rgtm1Wjl/SzJ) mice to generate liver metastases. Following metastatic tumour growth and humane endpoint criteria, mice were euthanised and their livers harvested. Tumour-bearing liver tissue (KP3) was either sectioned for blinded histopathological classification or used for protein extraction, to allow kinase profiling of these tumours. This design enabled a direct comparison between patient biopsy, PDO and PDO-derived tumour samples across both morphological and signalling dimensions.

**TABLE 1:** Comparison of Histopathological Features in Original Patient Metastatic Tumours (KP1) and Corresponding Mouse Xenografts (KP3). Summary of key morphological and cellular features observed in metastatic human tumour biopsies and matched xenograft tissues. The comparison highlights the preservation of tumour phenotype across models.

Sample	Patient tumour type	Biopsy collection site	Patient Biopsy (KP1)	Xenograft tumour (KP3)
#1	Metastatic colon/rectum adenocarcinoma	Liver	Metastatic adenocarcinoma from colon/rectum; collected from liver	Adenocarcinoma, high-to-moderate differentiation
			Glandular structures with luminal necrosis	Gland-like nodules with necrotic centers
			High cellular atypia	Eosinophilic cytoplasm, enlarged nuclei, desmoplasia, sparse angiogenesis
#2	Metastatic breast ductal carcinoma	Liver	Metastatic ductal carcinoma from breast; collected from liver	Adenomatous carcinoma, moderately diff entiated
			Solid and tubular structures	Nodular pattern, tumour cells are arranged in small to middle-size ducts (gland-like)
			Moderate pleomorphism, atrypical mitoses, desmoplastic stroma	Eosinophilic cytoplasm, enlarged nuclei; abnormal mitoses, central necrosis, desmoplasia, sparse angiogenesis
			HER2 overexpression	
#3	Metastatic cervical cancer	Pelvis	Metastatic cervical cancer; collected from pelvis	Squamous cell carcinoma, moderate differentiation: Nodular liver growth; necrosis, eosinophilic cytoplasm, enlarged nucle with enlarged nucleoli, abnormal mitoses, koilocytotic atypia, perinuclear halos, monocellular parakeratosis suggesting cervical cancer of HPV-related origin
			Inconclusive biopsy due to minimal, crushed material: Rare epithelial-like cells, p 16-, limited CK staining, no mitotic activity	​
#4	Metastatic colon/rectum adenocarcinoma	Liver	Metastatic adenocarcinoma from colon/rectum: Collected from liver	Adenomatous carcinoma, low-to-moderate differentiation
			Glandular tumour with necrosis; morphologically compatible with colorectal origin	Glandular structures. Necrosis, eosinophilic cytoplasm, enlarged nuclei, abnormal mitoses, desmoplasia, sparse angiogenesis
#5	Metastatic colon/rectum adenocarcinoma	Pleura	Metastatic adenocarcinoma from colon rectum; collected from the pleura	Adenocarcinoma, moderate differentiation
			Glandular structures, luminal necrosis; tumour ∼10% of sample, necrosis −30%, desmoplastic stroma	Nodular pattern, duct-like structures: ∼25% necrotic with mucin-like content (mucin production not ruled out): Eosinophilic cytoplasm, desmoplasia is rare

**FIGURE 2:**
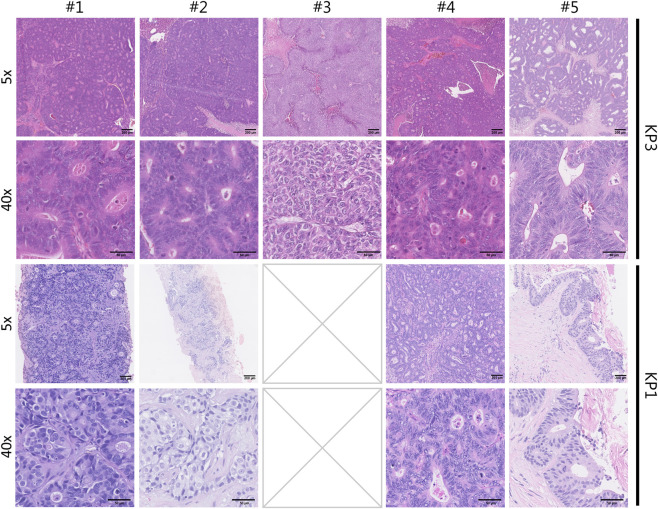
Hematoxylin and eosin (H&E) staining of patient biopsy and matched implanted PDO cancer cells in mouse liver tissue at low and high magnification. Representative histological images of the KP3 generated from the different patient derived organoids, as well as the matched H&E-stained section from the patients’ metastatic lesions (KP1), shown at ×5 magnification (top row) and ×40 magnification (bottom row). The top row illustrates the overall architecture and tumour growth within the liver parenchyma, while the bottom row highlights cellular morphology and tumour features at higher resolution. All sections were stained with H&E. Scale bar corresponds to 200 µm for the 5x images and 50 µm for the 40x images. KP1 for #3 not shown due to insufficient amount of quality patient biopsy sample for histological assessment.

To assess whether KP3 conserved histopathological features from the original metastatic lesions in the patient (KP1), KP3 samples were collected, sectioned, and stained for H&E (Figure 2). These histological samples were then blinded and classified by a pathologist. This classification was later compared to the original histopathological report from KP1. A summarised version of these reports can be observed in Table 1.

Table 1 summarises and compares the key histological and cellular features observed in original diagnostic biopsies from metastases of five patients (KP1) with those found in corresponding mouse xenograft tumours (KP3). Shared tumour characteristics–including glandular architecture, nuclear pleomorphism, necrosis, mitotic activity, and stromal changes–are highlighted to demonstrate the degree of morphological concordance between the human and mouse samples. The comparison supports the fidelity of the xenograft models in replicating the original metastatic lesion histopathology across all analysed samples.

### 3.2 PDO-derived tumours retain patient-specific kinase tumour signalling

**FIGURE 3:**
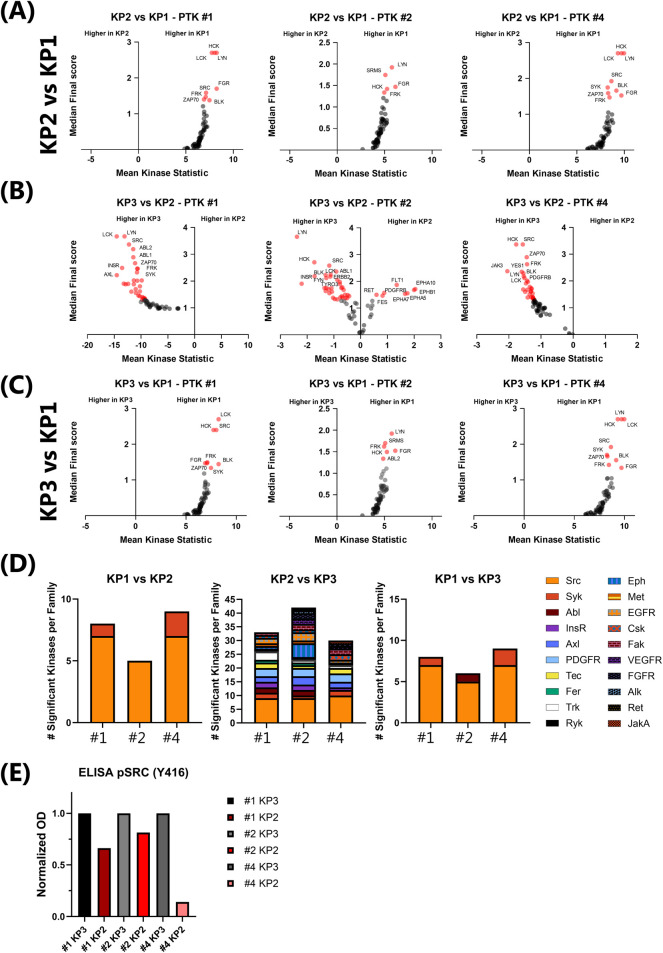
Kinase activity profiling of samples #1, #2 and #4 through Phosphotyrosine kinase (PTK) analysis. **(A–C)** Volcano plots illustrating differential tyrosine kinase activity between paired KP samples: **(A)** KP1 biopsies (right) and KP2 patient derived organoid lines (left); **(B)** KP2 (right) vs. KP3 (left); **(C)** KP1 (right) vs. KP3 (left). Kinase activity was inferred from phosphorylation patterns of tyrosine-containing peptide substrates using the PamGene PTK PamChip®. The x-axis represents the mean kinase statistic (reflecting average differential activity), while the y-axis shows the median final score, indicating confidence in kinase prediction. Kinase activity inference was performed using PamGene’s Tercen BioNavigator software. Significantly altered kinases are highlighted in red, revealing key signalling changes associated with tumour implantation. Kinases with a median final score ≥1.3 were considered significantly activated. Experiments were performed in triplicate for KP2 and KP3. Only one run was performed for the KP1 due to limited patient sample availability. **(D)** Frequency of significantly altered kinase families across samples. Kinases showing significant changes were filtered from the full dataset. The number of kinases belonging to each family was counted per sample, and the resulting frequencies were visualised as a barplot to illustrate the distribution of kinase family representation among significantly altered kinases between each comparison per matched sample. **(E)** Optical density values from PathScan® Phospho-Src (Tyr416) ELISA were normalised to matched KP3 PDOX samples (set to 1) for each patient case. KP2 PDOs are shown as fold-change relative to KP3.

To assess whether KP2 and KP3 faithfully reproduced the features of their metastatic tumour lesion of origin and to evaluate how kinase signalling changes across culture and *in vivo* contexts, we established matched sets of KP1, KP2 and KP3 samples for three of the patient derived samples: #2, #3 and #4, for which both the original metastatic biopsy and the matched xenograft tumours were obtained from the same anatomical site–the liver–thereby minimising site-specific variability in signalling. This selection minimised anatomical site–specific variability in kinase signalling and ensured direct comparability across biopsy, PDO, and PDOX samples. Kinase activity was inferred from proteomic data using the upstream kinase analysis (UKA) algorithm of the Bionavigator/Tercen softwares provided by Pamgene. Volcano-style plots show the distribution of the differential activity between samples, visualised for phospho-tyrosine kinases (PTKs) (Figure 3).

On a first instance, we compared the phosphotyrosine kinase profile of KP1 and KP2 in order to assess potential differences in kinase activity of these samples, when removed from a patient context and placed *in vitro* (Figure 3A). Across all cases, PTK activity profiling showed a significant decrease in the kinases activity of some on the KP2, particularly associated with the SRC-family of kinases (SFKs), which was amongst the most active family kinases across all samples (Figure 3D). Specifically, kinases such as HCK, LYN, FYN, FGR and FRK were significantly upregulated in the KP1 samples. This indicates a potential loss in SFKs when samples are removed from an *in vivo* context and placed *in vitro*.

Given this, we decided to look into whether this signature could be regained when we placed the KP2 samples back into an *in vivo* context. Therefore, we compared the kinase signature of KP2 to KP3 samples (Figure 3B).

Across all cases, PTK activity profiling showed that kinase activity was mainly increased in KP3 samples compared to KP2 samples, particularly associated with the SRC-family, which were once again amongst the most active family kinases across all samples (Figures 3B,D). Specifically, kinases such as SRC, LCK, HCK, FYN and BLK were significantly upregulated in the KP3 samples. Other kinase families (e.g., SYK, ABL, AXL, ISNR) showed variability in a case dependent matter, but SRC-family kinases were the most consistently altered (Figure 3D), underscoring their potential role as microenvironment-sensitive signalling hubs (Playford and Schaller, 2004; Lamar et al., 2019). Increased levels of activated Src (pSrc Y416) in KP3 samples compared to KP2 was further confirmed through ELISA (Figure 3E).

In case #2, the KP2 samples showed an increase in the activity of additional PTKs, including FLT1, RET, PDGFRA, and Ephrins when compared to KP3. These observations might reflect a transient adaptation to *in vitro* culture conditions, namely, the addition of growth factors known to interfere with these pathways (Fabro et al., 2022; Duong-Ly and Peterson, 2013; Radu and Chernoff, 2017). Regardless, an elevated SRC-family kinase activity was still observed in KP3, reinforcing the notion that distinct kinase programs are differentially modulated by culture versus grafted context, and suggesting case-dependent differences in microenvironment reprogramming. These kinases are known mediators of microenvironmental signalling, including integrin engagement, cytoskeletal remodelling, and interactions with stromal components (Al-Ejeh et al., 2014; de Bous et al., 2020; Yao et al., 2024; Kjølle et al., 2026; Rafaeva et al., 2022).

Interestingly, PTK analysis of KP1 vs. KP3, revealed that SRC-family kinases already presented higher active in the original patient metastases biopsies (KP1) (Figure 3C), suggesting that this activity was temporarily diminished in the PDOs (KP2), and partially reactivated upon *in vivo* engraftment in KP3 (Figure 3B). This pattern suggests that SFK signalling is context-dependent and sensitive to the presence of *in vivo* microenvironmental cues, which are lost in organoid culture but partially restored in the xenograft setting. These findings highlight the limitations of *in vitro* models in capturing microenvironment-sensitive signalling pathways and support the utility of KP3 models to uncover in vivo–dependent kinase activity that may have implications for drug response and therapeutic targeting. However, it is to note that the majority of the kinases analysed were not significantly altered, showing that 91%–95% of the approximately 100 kinases analysed were not significantly changed between KP2 and KP3, when compared to KP1.

Nevertheless, these results suggest that tumour intrinsic signalling is highly conserved between KP1, KP2 and KP3 samples, reinforcing this a robust model for personalised medicine and oncology research, while highlighting the importance of finding strategies that take into consideration the tumour microenvironment.

## 4 Discussion

In this study, we demonstrated that PDOs can remarkably retain and recapitulate histological and signalling features of their original metastatic tumour lesion when grafted into an animal model. Furthermore, we demonstrate that the microenvironment-sensitive signalling pathways partially lost in the *in vitro* setting are partially restored once placed in an *in-vivo* setting.

Using matched patient biopsies (KP1), PDOs (KP2) and PDO-derived mouse tumours (KP3), we performed blinded histological analyses and kinase activity profiling to evaluate how tumour architecture and signalling dynamics evolve across these model systems. Blinded pathological assessment revealed that the KP3 samples maintain key histological features of the original patient metastatic tumour lesions, supporting the notion that PDO models preserve tumour identity after *in vivo* engraftment. This reinforces the utility of these models as preclinical models and its relevance in in vivo studies.

Our kinase activity profiling uncovered a striking and consistent SRC-family of kinases (SFK) signature present *in vivo*, which is downregulated in PDO cultures compared to patient biopsies, but that is partially rescued upon *in vivo* implantation, suggesting that SFK activity is closely related to the tumour microenvironment, as already demonstrated through several studies (Playford and Schaller, 2004; Lamar et al., 2019; Que et al., 2025; Zhang et al., 2021; Zhao et al., 2025).

SFKs are key mediators of integrin signalling, stromal interactions, perception of biomechanical cues, cytoskeletal remodelling and crosstalk between tumour cells and other components of the tumour microenvironment (Katoh, 2024a; Playford and Schaller, 2004; Lamar et al., 2019; Que et al., 2025; Ortiz et al., 2021; Katoh, 2024b; Wang et al., 2011; Calvo et al., 2013; Li et al., 2021; Yui et al., 2018) – features that are inherently absent in 2D settings, and, in this case, organoid culture systems. The partial restoration of these signalling pathways in PDO-derived tumours implies that even a simplified *in vivo* niche, in this case provided by the immunodeficient NSG mice, is sufficient to re-engage key components of this microenvironment-associated signalling.

One of our cases, specifically #2, showed elevated activity of several receptor tyrosine kinases (RTKs), such as RET, FLT1, PDGFRB, and Ephrins in the PDO context. These could be linked to the growth factor supplementation present in organoid media, which are known to induce RTK expression and promote Wnt-Eph signalling crosstalk. Wnt signalling is known to upregulate Ephrins, especially in stem/progenitor contexts (Pasquale, 2024; Tanaka et al., 2003; Takahashi and Shiraishi, 2020). We did not experimentally test the effects of withdrawing specific growth factors, supplementing defined integrin-binding ECM ligands, or implementing stromal co-culture systems (for example, with fibroblasts), so we cannot directly determine how such modifications would modulate signalling in KP2. However, given the strong association of SRC-family kinases with integrin engagement and stromal interactions, our data support the hypothesis that ECM composition and co-cultured stromal cells would further influence these context-dependent signalling programs, and we highlight this as an important direction for future mechanistic work.

An additional consideration is the relative tumour cell content across KP1, KP2, and KP3. Parallel analyses in related cohorts indicate that PDO cultures (KP2) typically exhibit the highest proportion of tumour cells, whereas patient biopsies (KP1) and PDO-derived tumours (KP3) contain a variable admixture of tumour and stromal components. This difference in tumour cellularity should be taken into account when interpreting kinase activity profiles. In this context, complementary assessment of serine/threonine kinase activity would also be highly relevant and may further refine our understanding of context-dependent signalling changes.

In addition to these observations, we also saw patient-specific kinase responses across the different samples, also typical of the inherit heterogeneity characteristic of patient samples. These observations underscore the inherent heterogeneity in tumour signalling and highlight the importance of using matched, individualised models to capture patient-specific biology.

These differences suggest that the degree and nature of microenvironmental influence on kinase signalling may vary by tumour type, with breast and colorectal cancers potentially engaging distinct pathways in response to *in vitro* versus *in vivo* environments. Importantly, KP1 and KP3 contain stromal cells and other microenvironmental components apart from the tumour cells, which lack in KP2, which only contain cancer cells. This highlights the importance of considering tumour context when interpreting kinase activity profiles and drug responses in organoid-based models.

## 5 Conclusion

Taken together, our results reinforce the value of using precision medicine approaches to recapitulate human patient disease. Strikingly, isolation of cancer cells alone in PDO cultures under very non-physiological levels of oxygen, etc., recapitulates the majority of the signalling observed in the original tumour biopsy. Even more striking is that subsequent growth of the PDOs as liver metastases in mice restores almost identical signalling to the original metastatic tumour and even displays the majority of the histological features.

Our findings emphasise the limitations of PDOs as isolated systems for studying microenvironment-dependent signalling, while also validating PDO-derived tumour models as an intermediate that restores some, but not all, aspects of *in vivo* signalling architecture. This has direct implications for therapeutic screening: drugs targeting pathways like SFKs may appear inactive in PDOs yet remain effective *in vivo*, depending on microenvironmental context. Therefore, incorporating xenograft systems and/or more complex *in vitro* models that include other microenvironmental components can enhance the physiological relevance of functional precision medicine pipelines, thereby improving the predictive accuracy of preclinical drug testing.

Future studies should focus on further characterising the cellular and extracellular components responsible for SRC-family reactivation in PDO-derived tumours, including contributions from mouse stroma, vasculature, or mechanical cues. Co-culture systems and engineered matrices could offer complementary approaches to more fully reconstitute microenvironmental signalling *in vitro*. Ultimately, integrating proteomic, genomic, and spatial data across matched models will enable a more comprehensive understanding of how the tumour microenvironment shapes therapy response.

## Data Availability

The original contributions presented in the study are included in the article/Supplementary Material, further inquiries can be directed to the corresponding author.
